# M. Dupuytren on Union by the First and Second Intentions

**Published:** 1830-07-01

**Authors:** 


### HOTEL DIEU.

M. Dupuytren's Sentiments on Union by the First and Second Intention.*

It may be interesting, perhaps instructive, to British surgeons to hear the opinions of so eminent a Frenchman as M. Dupuytren, on the subject of union by the first intention. We know there are some gentlemen in this country who conceive their practice to be as near as possible to perfection, and measure the errors of their neighbours by the scale of distance from their own standard. Truth, however, is not so exclusive as prejudice, error is first cousin to bigotry, and all candid men will consider what is said on both sides of a question, before they decide the issue. The following remarks are extracted from the clinical lectures of M. Dupuytren.

It would appear that several amputations of the upper or lower extremities were performed last Winter at the Hôtel Dieu, and afforded the surgeon of that establishment an opportunity of delivering his sentiments and detailing the results of his experience. The ancient surgeons did not argue the advantages, or otherwise of union by the first intention, because by their modes of operating and dressing they could not well obtain it. The mode of procuring that desirable event was discovered in England, adopted with ardour in Germany, and, although employed by Dessault, condemned in the first instance,

* Journ. Hebdomad. No. 75. Mars, 1830.

and even now but half understood and reluctantly practised in France. The military surgeons of that country, who be it remembered were most frequently in contact with the English, defended the new method by word and by deed, and the benefits it promised seduced very many of the French Savans. Isolated cases of success poured in, but as M. Dupuytren very justly remarks, stray facts are merely chaff and bran, matters of no weight and arguments of little value in determining a great question. It is only by cases en masse, by the collection and comparison of a *number* of facts, that we can arrive at general and sound conclusions. "Now such a method of examination is unfavourable to the triumph of union by the first, over union by the second intention."

M. Dupuytren is convinced that more amputation patients are lost by the new than by the ancient method, and on a careful consideration of a number of cases he finds that the results are in favour of the latter. Out of thirty amputations of limbs (large and small) in which union by the second intention was attempted, there were six fatal cases; whilst out of twenty-nine other amputations in which union by the first intention was essayed, there were nine fatal cases. M. Dupuytren has on several occasions made similar calculations. The cases are mostly those of patients suffering from suppurations of long standing, as from caries, white swellings, &c. and it is impossible to arrest such discharges with suddenness and violence, without producing great disturbance in the system. Bold or insidious inflammations of the viscera are too often the consequences, and M. Dupuytren believes that phlebitis is more common under the present method than it was under the old one.

Union by the first intention succeeds better in the hands of military surgeons than in civil hospitals, because the subjects are active and vigorous men, who have not laboured previously under local inflammations or suppurations. Such subjects bear the operation better, and the symptoms that succeed are of a less complex and questionable nature, than those which occur in hospital patients. Persons in the latter who require amputation in consequence of accidental injuries, are cured as frequently as soldiers, and are as well adapted for union by the first intention. The advantages then of union by the first intention are far less brilliant, and its disadvantages more numerous than its partisans professed and believed. It is beginning to be abandoned, observes M. Dupuytren, in the country which gave it birth.

The able Baron himself appears to be decided on only attempting it in a few cases, and is resolved to adopt the old mode of dressing in the great majority. M. Dupuytren, however, has no intention of resuscitating the faults of the old system; of cramming the stump with lint and dressings, and tearing them away before they were properly detached by the suppurative process; a practice which did an infinity of mischief, and rendered the 'first dressing' more excruciating and dreadful than the operation itself. M. Dupuytren's intention is merely to interpose a certain quantity of fine lint between the sides of the stump, to approximate the latter gently by adhesive straps, and finally not to withdraw the dressings until they are loosened and cast off by the suppuration. By these means a more or less copious secretion of pus is kept up on the face of the stump, and the patient is not subjected to the inconveniences and dangers arising from the sudden stoppage of an habitual drain.

Such are the sentiments of a distinguished surgeon, and very shrewd observer of the thousands of facts which his situation at the Hôtel Dieu may be said to lay at his feet. A hospital like that is a kind of *harem*, wherein are collected, not the dark-eyed maids of Macedon or the Morea, nor the lovely forms of the Circassian, Georgian, and tenant of the Caucasus, but choice specimens of pathology, the beau idéal of the subjects of surgery. The most stupid man must reap *something* from such a crop of opportunities, an able one would lay up a store like Joseph's in Egypt. On these accounts the sentiments of M. Dupuytren deserve to be treated with deference, although they may not square with the experience of this country.

But still it is singular that union by the first intention should fail so much oftener in France than it does in England, and so many patients die in the attempt to procure it. We lately conversed with a gentleman, on whose accuracy we can place the most entire confidence, and who passed 15 months in the French metropolis for the purpose of attending at the Hôtel Dieu, and witnessing the practice of M. Dupuytren. He was present at the clinical lecture of which we have given an account, and saw the amputations on which that clinique was immediately founded. His sentiments are very unfavourable to the French mode of dressing the stumps, and lead us to believe that M. Dupuytren is deciding against a practice, the merits of which he never *properly* tested. The stumps, says our informant, are loaded with dressings, and on the slightest occasion they are all removed, and lint crammed into the wound to encourage suppuration. M. Dupuytren appears to have never been cordially attached to the English method, and we know that insincere attachment is apt to lapse into hostility on trifling grounds of offence, to magnify faults, and be blind to beauties. If the results of union by the first and by the second intention were examined in this country in the manner which M. Dupuytren recommends, namely, by numerical calculations, we more than suspect that the balance of success would be greatly in favour cf modern surgery. We shall return to this subject on another opportunity.

